# Artificial Teeth Worn by Washington

**Published:** 1842-09

**Authors:** 


*Artificial Teeth Worn by Washington.*—It is known to many that General Washington lost most of his natural teeth at an early age, and that for a number of years previously to his death he used artificial ones. The late Mr. John Greenwood, for a long time a distinguished dentist of New York, inserted, we believe, some two or three complete double sets for him. They were manufactured from the ivory of the tusk of the hippopotamus, almost the only material at that time employed for artificial teeth. We have half of a beautifully carved upper set, that were worn for several years in connection with a lower set, by General Washington, which were presented to us by Dr. Isaac I. Greenwood, son of the late Mr. John Greenwood, extensively known as an eminent practitioner of dental surgery of the city of New York. Mr. J. Greenwood's initials, which were engraved upon these teeth at the time of their fabrication, together with part of the date, filled with tartar or salivary calculus while they were worn, are now perfectly distinct.

Although the art of the dentist has made rapid advances since the time when these teeth were inserted, their execution is superior to that of many manufactured at the present day. They were secured in the mouth by means of spiral springs. The other half of the upper, and the whole of the lower set, are in the possession of Dr. Isaac I. Greenwood. H.

